# Supplementary material for: Infants at high and low likelihood for autism show different EEG developmental trajectories in speech tracking and statistical learning
Source: eLife. 2026 Sep 21;14:RP109901. doi: 10.7554/eLife.109901 (PMC13592813; doi:10.7554/eLife.109901)
Supplement: Peer review history

# Editor report: eLife Assessment

**DOI:** 10.7554/eLife.109901.3.sa0

**Authors:** Jean-Paul Noel

**Affiliations:** University of Minnesota United States

**Keywords:** Convincing, Important

This **important** study uses longitudinal EEG to chart how neural tracking of syllables and word-level statistical structure develops over the first two years of life in infants at high and low likelihood for autism and links these measures to verbal outcomes at 18-20 months. The strength of evidence is **convincing**: the prospective longitudinal design, careful data-quality handling, and partial least squares analyses are appropriate and well executed, though some interpretations of the group differences in syllable tracking, along with the possible contributions of multilingual exposure and sleep state during recording, warrant caution. The work will be of interest to developmental cognitive neuroscientists studying language acquisition and early neural markers of neurodevelopmental conditions.

# Referee report: Reviewer #1 (Public review)

**DOI:** 10.7554/eLife.109901.3.sa1

Summary:

This manuscript reports a prospective longitudinal study examining whether infants with high likelihood (HL) for autism differ from low-likelihood (LL) infants in two levels of word learning: brain-to-speech cortical entrainment and implicit word segmentation. The authors report reduced syllable tracking and post-learning word recognition in the HL group relative to the LL group. Importantly, both the syllable-tracking entrainment measure and the word recognition ERP measure are positively associated with verbal outcomes at 18-20 months, as indexed by the Mullen Verbal Developmental Quotient. Overall, I found this to be a thoughtfully designed and carefully executed study that tackles a difficult and important set of questions. With some clarifications and modest additional analyses or discussion on the points below, the manuscript has strong potential to make a substantial contribution to the literature on early language development and autism.

Strengths:

This is an important study that addresses a central question in developmental cognitive neuroscience: what mechanisms underlie variability in language learning, and what are the early neural correlates of these individual differences? While language development has a relatively well-defined sensitive period in typical development, the mechanisms of variability-particularly in the context of neurodevelopmental conditions-remain poorly understood, in part because longitudinal work in very young infants and toddlers is rare. The present study makes a valuable contribution by directly targeting this gap and by grounding the work in a strong theoretical tradition on statistical learning as a foundational mechanism for early language acquisition.

I especially appreciate the authors' meticulous approach to data quality and their clear, transparent description of the methods. The choice of partial least squares correlation (PLS-c) is well motivated, given the multidimensional nature of the data and collinearity among variables and the manuscript does a commendable job explaining this technique to readers who may be less familiar with it.

The results reveal interesting developmental changes in syllable tracking and word segmentation from birth to 2 years in both HL and LL infants. Simply mapping these trajectories in both groups is highly valuable. Moreover, the associations between neural indices of brain-to-speech entrainment and word segmentation with later verbal outcomes in the LL group support a critical role for speech perception and statistical learning in early language development, with clear implications for understanding autism. Overall, this is a rich dataset with substantial potential to inform theory.

Comment on revised version.

The revised manuscript has provided additional analyses that lead to critical clarification of the main findings, including the longitudinal nature of the relationship between neural tracking of speech and language, the role of sleep, and the potential modulation effect of stream structure on syllable-level neural tracking. The overall results highlight the robustness of the findings as well as the specific relevance of the structured speech tracking to verbal outcomes of infants with high likelihood (HL) of autism.

# Referee report: Reviewer #2 (Public review)

**DOI:** 10.7554/eLife.109901.3.sa2

Summary:

This article looks at differences in how the brain entrains to, or tracks, the rhythmic presentation of syllables and words in speech in infants at increased likelihood versus low likelihood for autism. The authors first sought to characterize how brain responses are modulated by learning the statistical probability of a given syllable following the one before it over the first two years of life. They then sought to identify at which stages of word learning infants at increased likelihood for autism showed difficulties, and whether those difficulties worsened over time. Finally, they sought to indicate whether infants' statistical learning and word learning abilities could predict later verbal skills. The authors found similar developmental trajectories of neural entrainment to syllables in infants at high and low likelihood for autism, but infants at high likelihood for autism had overall weaker syllable-level entrainment. Infants at high versus low likelihood for autism showed different developmental trajectories for word entrainment. Lower syllable entrainment in high-likelihood infants corresponded with poorer verbal outcomes, but word entrainment was not associated with verbal outcomes. Event-related potential responses to words and part words were positively associated with verbal outcomes, however, but only in low-likelihood infants.

Strengths:

Overall, the article provides rigorous statistical analysis of longitudinal EEG data to provide strong support for the claims that neural entrainment to syllable and word features of speech may be a useful marker for language development difficulties, particularly in infants at increased likelihood for neurodevelopmental disorders. The EEG data collection and preprocessing procedures are well within standards within the field. Readers should take care to note that authors indexed neural entrainment to speech using phase-locking values instead of spectral power.

Comments on revised version.

While the statistical analyses are rigorous, there are a few potential confounds to the results. The authors now do a nice job addressing these limitations to the work. For example, sleep status may modulate some of the biomarkers relevant for language learning. Exposure to additional languages may influence performance on the verbal assessment, though the authors do clarify that participants came from majority French-speaking households. As a result, readers should be encouraged to interpret that neural entrainment to speech features is likely a useful mechanism to explain differences in language development, while taking this interpretation with some caution.

# Author comment: Author response

**DOI:** 10.7554/eLife.109901.3.sa3

**Authors:** Michel Godel, Ana Fló, Lucas Benjamin, Ghislaine Dehaene-Lambertz, Marie Schaer

**Affiliations:** University of Geneva Geneva Switzerland; University of Padua Padua Italy; Université Paris-Saclay Paris France; Université Paris- Saclay Paris-Saclay France; University of Geneva Geneva Switzerland

The following is the authors’ response to the current reviews.

**Reviewer #1 (Recommendations for the authors):**

(1) Interpretation of Syllable-Tracking in the RND Condition:

The finding of greater syllable-tracking in the LL group compared to the HL group in the RND condition warrants cautious interpretation. Currently, there is no direct statistical evidence demonstrating greater PLV at 4 Hz in the Structured versus Random conditions for either group; readers must infer this solely from numeric differences in Figure S5 B and D. Therefore, while the interpretation on Page 14 (Lines 443-446) "successful segmentation may enhance syllable tracking via top-down predictions of the next syllable" is an interesting speculation, it feels somewhat far-reaching. Additionally, the authors should discuss whether this upregulated syllable tracking in the structured condition (which is specific to the HL group) represents an adaptive or maladaptive response.

The reviewer correctly highlights the lack of direct comparison between conditions (RND versus STR). We tempered our claims in the cited paragraph and insisted on the speculative nature of this part of the discussion. We also clarified that, to us, it may represent an adaptive compensatory strategy:

Page 14, line 441: “Interestingly, our supplementary analyses (Supplementary Material Figure S4-5) suggest that syllable entrainment may be differentially affected in HL versus LL infants, depending on the statistical structure of the input stream (RND versus STR). However, as our experiment was not explicitly designed to test stream effects, these results should be interpreted with caution. Future studies could explore how successful segmentation may enhance syllable tracking via top-down predictions of the next syllable in both LL and HL infants. If confirmed, such a mechanism may improve alignment to syllable onsets, potentially constituting a compensatory process allowed by preserved segmentation abilities.”

(2) Preservation of Statistical Learning in HL Infants:

The text added on Pages 17-18 (Lines 562-566) regarding a "heightened dependence on bottom-up mechanisms (in autism)" does not appear to be supported by the data or by theories of implicit statistical learning. Because greater syllable-level entrainment was observed in the LL group than the HL group across both the random and structured conditions, the data actually point toward impaired bottom-up processes. Furthermore, implicit statistical learning typically involves an interplay of both bottom-up and top-down mechanisms; the implicit nature of a task does not guarantee a strictly bottom-up process. Consequently, this interpretation is not entirely convincing.

We agree with the reviewer that the concepts of “top-down” and “bottom-up” were not fully appropriate to support our point in the cited paragraph. We should have used the concepts of implicit versus explicit learning instead, in line with previous literature suggesting increased reliance on preserved implicit learning in autism to compensate for altered explicit processes. The paragraph was slightly modified.

Page 18, line 564: “According to these studies, autistic impairments in explicit attentional processes, such as social orienting - which are critical for bootstrapping language acquisition (70) - may result in a heightened dependence on implicit mechanisms, including statistical learning. As previously discussed, preserved word segmentation abilities may further compensate for alterations in lower-level implicit processes, such as syllable tracking.”

**Reviewer #2 (Recommendations for the authors):**

Potential typo on line 199 - I think an apostrophe is needed here.

Potential typo on line 255 - do you mean Central electrodes?

We addressed the typos spotted by reviewer.

Line 199: variables’

Line 255: Centro-frontal electrodes

The following is the authors’ response to the original reviews

**Public Reviews:**

**Reviewer #1 (Public review):**

Summary:

This manuscript reports a prospective longitudinal study examining whether infants with high likelihood (HL) for autism differ from low-likelihood (LL) infants in two levels of word learning: brain-to-speech cortical entrainment and implicit word segmentation. The authors report reduced syllable tracking and post-learning word recognition in the HL group relative to the LL group. Importantly, both the syllable-tracking entrainment measure and the word recognition ERP measure are positively associated with verbal outcomes at 18-20 months, as indexed by the Mullen Verbal Developmental Quotient. Overall, I found this to be a thoughtfully designed and carefully executed study that tackles a difficult and important set of questions. With some clarifications and modest additional analyses or discussion on the points below, the manuscript has strong potential to make a substantial contribution to the literature on early language development and autism.

Strengths:

This is an important study that addresses a central question in developmental cognitive neuroscience: what mechanisms underlie variability in language learning, and what are the early neural correlates of these individual differences? While language development has a relatively well-defined sensitive period in typical development, the mechanisms of variability - particularly in the context of neurodevelopmental conditions - remain poorly understood, in part because longitudinal work in very young infants and toddlers is rare. The present study makes a valuable contribution by directly targeting this gap and by grounding the work in a strong theoretical tradition on statistical learning as a foundational mechanism for early language acquisition.

I especially appreciate the authors' meticulous approach to data quality and their clear, transparent description of the methods. The choice of partial least squares correlation (PLS-c) is well motivated, given the multidimensional nature of the data and collinearity among variables, and the manuscript does a commendable job explaining this technique to readers who may be less familiar with it.

The results reveal interesting developmental changes in syllable tracking and word segmentation from birth to 2 years in both HL and LL infants. Simply mapping these trajectories in both groups is highly valuable. Moreover, the associations between neural indices of brain-to-speech entrainment and word segmentation with later verbal outcomes in the LL group support a critical role for speech perception and statistical learning in early language development, with clear implications for understanding autism. Overall, this is a rich dataset with substantial potential to inform theory.

Weaknesses:

(1) Clarifying longitudinal vs. concurrent associations

Because the current analytical approach incorporates all time points, including the final visit, it is challenging to determine to what extent the brain-language associations are driven by longitudinal relationships vs. concurrent correlations at the last time point. This does not undermine the main findings, but clarifying this issue could significantly enhance the impact of the individual-differences results. If feasible, the authors might consider (a) showing that a model excluding the final visit still predicts verbal outcomes at the last visit in a similar way, or (b) more explicitly acknowledging in the discussion that the observed associations may be partly or largely driven by concurrent correlations. Either approach would help readers interpret the strength and nature of the longitudinal claims.

We thank the reviewer for this insightful comment. We agree that distinguishing between longitudinal predictive power and concurrent correlations at the final visit is crucial for clarifying the nature of these brain-language associations. Following the reviewer’s suggestion (a), we re-ran the two critical Partial Least Squares Correlation (PLS-c) analyses by excluding all EEG and behavioral data from the final 18–21 month visit (n = 54 recordings kept) to test whether earlier trajectories still predict the final verbal outcome.

(1) Syllable entrainment (4 Hz) (original analysis on Figure 2C–D): The PLS-c restricted to the 3- to 15-month visits still identified a single significant component (p=.001, r=.56, 64.0% explained covariance, Figure 2 -figure supplement 3). Bootstrap ratios (BSR) were: contrast (low vs. high autism likelihood) 4.1; mean age −1.3; contrast*mean-age −2.1; delta-age 10.5; contrast*delta-age 2.0; age^2^ −6.0; contrast*age^2^ −5.8; and notably verbal outcome 7.1; contrast*verbal-outcome −6.3.

The latent component and its spatial electrode configuration remain highly consistent with the original analysis (Figure 2C–D). This confirms that excluding the final visit preserves the model’s predictive validity: lower syllable entrainment correlates with poorer verbal outcomes at 18–21 months, particularly in the high-likelihood group.

(2) Late evoked response to novel words (original analysis on Figure 6): The PLS-c analysis on the ERP late time window (1500–3000 ms), excluding the final visit, also revealed one significant component (p=.002, r=.74, 33.5% explained covariance, Figure 6 -figure supplement 1). Bootstrap ratios (BSR) were: contrast (part-word versus word) 14.5; mean age -5.2; contrast*mean-age 6.2; delta-age -0.8; contrast*delta-age 3.5; age^2^ -0.8; contrast*age^2^ -12.1; verbal-outcome 20.1; contrast*verbal-outcome -7.2. The latent component closely mirrored the original analysis (Figure 6), with frontal electrodes contributing negatively and posterior electrodes positively. Minor divergences in age-related parameter contributions were observed, likely due to the absence of 18-21 month timepoints, which previously contributed to the convex/concave shapes of the group age trajectories in figure 6B (left panel).

Crucially, both models (with and without the final visit) positively predicted verbal outcomes (Figure 6B, right panel, and Figure 6 -figure supplement 1B). However, excluding the final visit reversed the direction of the group*verbal-outcome interaction (from 3 to -7.2): This indicates that after ruling out cross-sectional correlations at 18–21 months, the early predictive value of the late ERP to word novelty is more prominently observed in high-likelihood infants, suggesting that the original result was influenced by concurrent cross-sectional correlations at the final visit. This aligns with the syllable entrainment findings (Figure 2 -figure supplement 3), as both 4 Hz neural tracking and late ERP responses to novelty predominantly predict verbal outcomes in infants at high likelihood for autism.

We reported these supplementary analyses in the revised manuscript as follows:

We added Figure 2 -figure supplement 3 and Figure 6 -figure supplement 1. In general, most of figures that were present in Supplementary materials were moved as figure supplements to enhance readability.

Page 8 lines 234-240 (pages and lines refer to the reviewed uploaded manuscript): “To rule out the possibility that the association between syllable entrainment and verbal outcome was driven by concurrent measures taken at 18–21 months, we re-ran the PLS-c analysis excluding EEG data from the final visit (n = 54 recordings kept). The resulting latent component remain significant (p = .001) and showed contributions from behavioral and EEG variables that were highly similar to those observed in the previous analysis, with a verbal outcome BSR of 7.1 and a group’verbal-outcome interaction BSR of −6.3 (Figure 2 -figure supplement 3).”

Page 12 lines 387-394: “As we did for neural entrainment to syllables, we conducted a new analysis on late ERP to word novelty, excluding EEG data from the final visit. This PLS-c yielded one significant latent component (p = .002, r=.74, 33.5% explained covariance, Figure 6 -figure supplement 1) with globally similar EEG parameter contributions and age trajectory modelling. Verbal outcome still significantly contributed to the latent component (BSR=20.1), with a negative verbal outcome*group interaction (BSR=-7.2). These results suggest that, after ruling out cross-sectional correlations at 18–21 months, the late ERP to word novelty predominantly predicts verbal outcomes in high-likelihood infants for autism.”

Page 17 lines 547-548: “As with syllable entrainment, the late ERP to novel words primarily predicted verbal outcomes in high-likelihood (HL) infants.”

Page 18 lines 588-590: “Likewise, the absence of a late ERP orientation response in HL participants may represent an early neural signature of altered attention to novelty that can be used both as a non-invasive predictor of language development and as a potential target for early intervention.’

(2) Incorporating sleep status into longitudinal models

Sleep status changes systematically across developmental stages in this cohort. Given that some of the papers cited to justify the paradigm also note limitations in speech entrainment and word segmentation during sleep or in patients with impaired consciousness, it would be helpful to account for sleep more directly. Including sleep status as a factor or covariate in the longitudinal models, or at least elaborating more fully on its potential role and limitations, would further strengthen the conclusions and reassure readers that these effects are not primarily driven by differences in sleep-wake state.

The reviewer is highlighting here a limitation of our study design that comprised sleeping status that varied from one timepoint to another among participants. To rule out any confounding effect of wake status (coded as a binary variable: sleeping or awake during recording) on analyses comparing groups, a linear mixed-effect model with repeated measures was fitted finding no significant difference between high- and low-likelihood participants (p=.769, reported at page 20, lines 646-647). However, as rightly suggested by the reviewer, this doesn’t prevent from a sleep bias on age trajectories, especially given that sleeping status significantly decreases with age in our sample.

Including sleep status as a covariate in our analyses, as suggested by the reviewer, would be difficult to implement in our PLS-c methods, since a categorical behavioral parameter that varies within participants is not possible in the models provided by myPLS toolbox.

As an alternative option, we re-ran all analyses that explored the condition effect on the whole sample within the sleeping participants only (n=25 recordings) to confirm that the same age-trajectories of EEG parameters were highlighted. However, negative results should be interpreted with caution since the sample is small for such a multivariate approach, resulting in modest statistical power.

(1) Syllable entrainment (4 Hz) (original analysis on Figure 2A–B): The PLS-c identified one significant component (p <.001, r = .78, 85.1% explained covariance, Figure 2 -figure supplement 2 and Figure 3 -figure supplement 1). Bootstrap ratios (BSR) were: contrast (4hz vs. adjacent frequencies) 30.3; mean age -2.9; contrast*mean-age -2.4; delta-age 3.8; contrast*delta-age 3.4; age^2^ -1.1; contrast* −2.5. The spatial distribution of contributing electrodes globally matched that shown in Figure 2A. The high contrast BSR (30.3) confirms robust syllable entrainment in sleeping infants. Critically, the contrast*age^2^ parameter contributed negatively to the latent component (BSR = −2.5), confirming that the convex age trajectory of syllabic entrainment (Figure 2B) is also present in the sleeping subsample.

(2) Word entrainment (1.3 Hz) (original analysis on Figure 3A–B): The PLS-c identified one significant component (p <.001, r = .63, 37.3% explained covariance, Author response image 1). Bootstrap ratios (BSR) were: contrast (1.3hz vs. adjacent frequencies) 24.9; mean age -7.5; contrast*mean-age -3.2; delta-age 2.9; contrast*delta-age 0.0; age^2^ 1.5; contrast* 1.1. The spatial distribution of significant electrodes partially overlaps with the ones in the original analysis, primarily showing fronto-central positive contribution to the latent component. The high contrast BSR confirms a robust word entrainment in sleeping participants, in line with previous studies (e.g., Flò et al, Sci Rep, 2022). However, the lack of a significant contrast* age^2^ suggests that the U-shape age trajectory illustrated on Figure 3 might be modulated by wakefulness or due to a lack of power in the present analysis. A non-significant trend towards a U-shape pattern with a 12-month nadir is visible in sleeping participants, but additional data from sleeping 18-21 months sleeping infants would be required to confirm or refute this trend.

(3) Early evoked response to novel words (original analysis on Figure 4): The PLS-c analysis on the ERP early time window (0–1000 ms) in sleeping participants revealed no significant component. The absence of early response to word novelty in sleeping participant might account for the lack of response observed in the whole sample, illustrated on Figure 4. To test this hypothesis, we conducted the same PLS-c in awake participants (n=58 recordings), which also yielded no significant latent component. This suggests that the lack of a measurable early response to word novelty observed in the whole sample is consistent across both sleeping and awake infants, and not driven by any of the two subsamples.

(4) Late evoked response to novel words (original analysis on Figure 5): The PLS-c analysis on the ERP late time window in sleeping participants revealed no significant component. This suggests that sleeping participants might present a reduced or even absent late response to novel words. Given this identified effect of sleep on late ERP response, we reran the PLS-c on the late ERP window using group as contrast (original analysis on figure 6), excluding the sleeping participants to avoid any confounds. This PLS-c revealed one significant component (p = .006, r = .68, 30.5% explained covariance, Author response image 1). Bootstrap ratios (BSR) were: contrast (low versus high likelihood) 7.6; mean age -5.9; contrast*mean-age -3.8; delta-age 2.5; contrast*delta-age -3.5; age^2^ -2.5; contrast*age^2^ -4.9; verbal-outcome 8.5; contrast*verbal-outcome -1.0. Behavioral parameters contribute to this latent component with similar magnitude and polarity as in the original analysis. Electrode contributions are also highly consistent, with frontal negative and posterior positive contributions. This confirms that sleeping participants, despite their potentially reduced late response, did not significantly bias the results presented in Figure 6.

**Author response image 1.** Late evoked response potential (ERP) to word novelty in awake participants. A. Design and brain saliences derived from the significant latent component. Brain topographies of bootstrap ratios (BSR) are displayed at 250ms intervals. Black dots indicate BSR > 2.3. B. Participants’ brain scores for part-word and word conditions, as a function of age (left panel) and verbal DQ (right panel). For details on brain scores, see Figure 6 -figure supplement 1. Linear fitting is used for illustrative purposes only. HL: high likelihood for autism; LL: low likelihood for autism.

We reported these analyses in the revised manuscript as follows:

We added Figure 2 -figure supplement 2A and Figure 3 -figure supplement 1.

Page 7, lines 218-224: “Because some infants were asleep during the recording session, particularly at younger ages, we performed a supplementary control analysis restricted to this sleeping subsample (n = 25 recordings, Figure 2 -figure supplement 2). This PLS-c also identified a significant latent component (p < .001, r = .78, 85.1% explained covariance), with a significant contrast effect (BSR = 30.3) and a significant negative contrast*age^2^ interaction (BSR = −2.5). These findings confirm that the convex age trajectory observed in the main analysis remains present and observable even in sleeping infants.”

Page 8, lines 252-259: “We further investigated word entrainment in sleeping participants (n=25), which yielded one significant latent component (p<.001, r=.63, 37.3% explained covariance, Figure 3 -figure supplement 1). Centro-frontal electrode contributed to this component, with a high contrast BSR (24.9), confirming a similar word entrainment pattern in the sleeping subsample. The contrast*age^2^ was also positive but not significant (1.1), suggesting a trend toward a U-shape age trajectory with a 12-month nadir in sleeping infants. Additional 18-21 month recording would be required to confirm this trend.”

Page 11, lines 347-349: “The same PLS-c, conducted separately in sleeping (n=25) and awake subsamples (n = 58), yielded no significant latent component, indicating a consistent absence of early response to word novelty in both sleeping and awake infants.”

Page 11-12 lines 368-370: “The same PLS-c in the sleeping subsample yielded no significant latent component, suggesting that sleep may reduce or even abolish the late response to word novelty.”

Page 12 lines 382-385: “Given that no late response was detected in sleeping participants, we re-ran the PLS-c analysis using group as a contrast in the awake subsample (n=58). This yielded one significant latent component (p=.006, r=.68, 30.5% explained covariance), with behavioral and electrode contributions highly overlapping with those in Figure 6.”

Page 18 lines 590-592: “This potential biomarker might nevertheless be modulated by participants’ sleep status, warranting careful consideration of vigilance state in future studies.”

(3) Use of PLS-c and potential group × condition interactions

I am relatively new to PLS-c. One question that arose is whether PLS-c could be extended to handle a two-way interaction between group and condition contrasts (STR vs. RND). If so, some of the more complex supplementary models testing developmental trajectories within each group (Page 8, Lines 258-265) might be more directly captured within a single, unified framework. Even a brief comment in the methods or discussion about the feasibility (or limitations) of modeling such interactions within PLS-c would be informative for readers and could streamline the analytic narrative.

The reviewer raises a valid concern regarding the capacity of PLS-c to accommodate multi-way interactions among categorical and continuous variables. While PLS-c has no inherent theoretical constraints on the number of predictor terms (they can even exceed the sample size in number), practical limitations arise from model stability and interpretability when the ratio of predictors to sample size becomes excessive. As noted by Geladi and Kowalski (1986), exceeding ~10% of the sample size with predictors increases noise sensitivity and overfitting.

In our study, the PLS-c analyses already reach this ~10% limit, with a maximum of nine predictors for a sample size of n=83. Attempting to integrate both group and condition as contrasts — along with necessary age parameters to account for developmental trajectories — would result in 12 predictors (or 15 if verbal outcome is included). Specifically, the model would require behavioral terms for Group, Condition, Group*Condition, Mean-age, Group*Mean-age, Condition*Mean-age, Delta-age, Group*Delta-age, Condition*Delta-age, Age^2^, Group*Age^2^, Condition*Age^2^, Verbal-outcome, Group*Verbal-outcome, and Condition*Verbal-outcome.

Although a unified multivariate model capturing the complex dynamics at play in our sample is theoretically appealing, the substantial risk of overfitting precludes its feasibility. Therefore, we opted to use only one categorical predictor per PLS-c analysis to maintain model parsimony and reliability. However, a larger sample could overcome this limitation, allowing a stable and unified model of longitudinal EEG data that simultaneously captures age trajectories, group, clinical outcome, and condition.

Reference:

Geladi, P., & Kowalski, B. (1986). Partial least-squares regression: A tutorial. Analytica Chimica Acta, 185, 1–17. https://doi.org/10.1016/S0003-2670(00)82582-3

We added the following comment in the method section:

Page 24, lines 773-777: “We limited the number of behavioral variables to nine to mitigate noise sensitivity and overfitting risks associated with exceeding the 10% sample size threshold (Geladi & Kowalski, 1986). This limitation precluded the implementation of a single PLS-c model incorporating group, condition (STR vs. RND), age, and their interactions.”

(4) STR-only analyses and the role of RND

Page 8, Lines 241-245: This analysis is conducted only within the STR condition. The lack of group difference observed here appears consistent with the lack of group difference in word-level entrainment (Page 9, Lines 292-294), suggesting that HL and LL groups may not differ in statistical learning per se, but rather in syllabic-level entrainment. As a useful sanity check and potential extension, it might be informative to explore whether syllable-level entrainment in the RND condition differs between groups to a similar extent as in Figure 2C-D. In other work (e.g., adults vs. children; Moreau et al., 2022), group differences can be more pronounced for syllable-level than for word-level entrainment. Figure S6 seems to hint that a similar pattern may exist here. If feasible, including or briefly reporting such an analysis could help clarify the asymmetry between the two learning measures and further support the interpretation of syllabic-level differences.

The reviewer points to the interesting pattern highlighted in supplementary figure S6, suggesting that group differences in syllabic entrainment might be modulated by the structure of the stream (STR versus RND). Such modulatory effect of stream structure on entrainment to syllables has been suggested by many studies, like Moreau et al (2022), as pointed by the reviewer, and seems at play in our sample, as illustrated on supplementary figure S5 (decline in the 4hz PLV that exceeds the size of confidence intervals, ~90 s after STR onset).

Following the reviewer’s suggestion, we ran a PLS-c testing group effect on 4hz PLVs in each stream:

(1) in the RND stream: the analysis yields one significant component (p<.001, r=.49, 52.7% explained covariance, Author response image 2A-B). Bootstrap ratios (BSR) are: contrast (low versus high likelihood) 6.9; mean age -1.0; contrast*mean-age 0.1; delta-age 11.8; contrast*delta-age 0.1; age^2^ -4.9; contrast*age^2^ 4.6; verbal-outcome 9.7; contrast*verbal-outcome -0.6. Interestingly, the model still highlights a strong link between syllable tracking and group, suggesting that RND also discriminate between HL and LL. However, RND syllable tracking doesn’t appear to be linked to group x verbal-outcome as we observed in Figure 2C-D.

(2) In the STR stream, we obtained one significant latent component (p=.002, r=.51, 57.8% explained covariance, Author response image 2C-D). Bootstrap ratios (BSR) are: contrast 2.2; mean age -1.6; contrast*mean-age -1.1; delta-age 6.1; contrast*delta-age -0.7; age^2^ -4.4; contrast*age^2^ 0.5; verbal-outcome 8.2; contrast*verbal-outcome -7.3. Here, the strong association between syllable tracking and group x verbal-outcome is similar to the model presented in Figure 2C-D.

Taken together, these results suggest that the apparent STR/RND dissociation illustrated in Figure S6 might primarily reflect a Group*Verbal-outcome divergence, with syllable tracking in the STR stream being related to verbal outcome mainly in high likelihood for autism.

**Author response image 2.** Syllable entrainment within RND (A-B) and STR (C-D).

These results were reported in the revised manuscript in the Result section (Time course of the entrainment along experiment subheader), implying a slight reframing of the result presentation of supplementary analysis S6. Author response image 2 was added in supplementary material as Figure S5.

Page 10, lines 307-319: “The group, age and verbal outcome parameters were mainly correlated (BSR>2.3) with the neural entrainment occurring~90 seconds after the onset of the STR stream, coinciding with the time participants began tracking word boundaries (Supplementary material, S3). This result suggests that the group differences in syllable entrainment, as shown in Figure 2C-D, as their associations with verbal outcome, are modulated by the structure of the stream (STR versus RND). We ran one additional PLS-c for each stream separately, using group as contrast. In both streams, the PLS-c yielded a significant LC (p<.001 for RND and p=.002 for STR), with a positive group effect (BSR>2.3) in both LC (Supplementary material, S5). Most strikingly, the group*verbal outcome parameter reached significance exclusively within the STR latent component (BSR:-7.3). These results suggest that while syllable tracking is generally decreased in HL infants across both streams, its association with verbal outcome is prominently driven by the stream containing words (STR).”

Page 14, lines 443-446: “This temporal overlap suggests that successful segmentation may enhance syllable tracking via top-down predictions of the next syllable, improving alignment to syllable onsets in LL infants as well as in HL with better verbal outcome.’

(5) Multi-speaker input and voice perception (Page 15, Lines 475-483)

The multi-speaker nature of the speech input is an interesting and ecologically relevant feature of the design, but it does add interpretive complexity. The literature on voice perception in autism is still mixed: for example, Boucher et al. (2000) reported no differences in voice recognition and discrimination between children with autism and language-matched non-autistic peers, whereas behavioral work in autistic adults suggests atypical voice perception (e.g., Schelinski et al., 2016; Lin et al., 2015). I found the current interpretation in this paragraph somewhat difficult to follow, partly because the data do not directly test how HL and LL infants integrate or suppress voice information. I think the authors could strengthen this section by slightly softening and clarifying the claims.

We acknowledge the reviewer’s concern regarding the potential ambiguity in the cited paragraph. To address this, we have revised the text to explicitly clarify the aims of our study and its design. Furthermore, we now emphasize the speculative and post-hoc nature of the hypotheses and interpretations presented, thereby ensuring transparency regarding the limitations of our findings.

Page 16 lines (520-530), as follows: “HL infants, on the other hand, did not show this transient disruption. In this group, word entrainment remained stable over time. To account for this unexpected finding, we followed up on the post-hoc hypothesis proposed above: a reduced sensitivity to social and vocal cues observed in HL infants may have spared segmentation abilities by limiting the interference introduced by speaker variability. If this post-hoc hypothesis holds true, LL and HL infants would differ not in their intrinsic ability to learn statistical regularities per se, but rather in how they integrate or suppress competing cues (such as speaker changes) during the segmentation process. It is important to note, however, that the present study was not designed to isolate and evaluate the specific impact of speaker changes on word segmentation. Consequently, this interpretation remains speculative, and additional research is required to further address this question.”

(6) Asymmetry between EEG learning measures

Page 16, Lines 502-507 touches on the asymmetry between the two EEG learning measures but leaves some questions for the reader. The presence of word recognition ERPs in the LL group suggests that a failure to suppress voice information during learning did not prevent successful word learning. At the same time, there is an interesting complementary pattern in the HL group, who show LL-like word-level entrainment but does not exhibit robust word recognition. Explicitly discussing this asymmetry - why HL infants might show relatively preserved word-level entrainment yet reduced word recognition ERPs, whereas LL infants show both - would enrich the theoretical contribution of the manuscript.

We concur with the reviewer’s observation that our findings imply a theoretically significant double dissociation between HL and LL groups, specifically concerning the asymmetries between word-level neural entrainment and word recognition mechanisms. We believe this point was partly addressed in the subsequent paragraph, where we stated that “in contrast” to LL, HL infants “showed no clear ERP difference between novel and familiar triplets”, while “both groups showed similar word neural entrainment during learning”. We further explored potential explanations for this apparent dissociation, such as a possible deficit in novelty orientation that may be specific to HL infants and unrelated to statistical learning itself. We cited Liu et al (2023) as a reference showing the dissociation between mechanisms underlying implicit versus explicit traces of statistical learning. We acknowledge that we can discuss more in depth the potential preservation of statistical learning in HL infants. We have incorporated the following discussion in the reviewed manuscript, supported by relevant references:

Pages 17-18, lines 562-566: “Interestingly, this dissociation between spared implicit versus impaired explicit statistical learning in autism has been previously discussed in the literature (Zwart et al, 2018, Kissine, 2021). According to these studies, autistic impairments in top-down attentional processes, such as social orienting — which are critical for bootstrapping language acquisition (Kuhl, 2007) — may result in a heightened dependence on bottom-up mechanisms, including implicit statistical learning.”

References:

Zwart, F.S., Vissers, C.T.W.M., Kessels, R.P.C. and Maes, J.H.R. (2018), Implicit learning seems to come naturally for children with autism, but not for children with specific language impairment: Evidence from behavioral and ERP data. Autism Research, 11: 1050-1061. https://doi.org/10.1002/aur.1954

Kissine, M. (2021). Autism, constructionism, and nativism. Language 97(3), e139-e160. https://dx.doi.org/10.1353/lan.2021.0055.

Kuhl, P.K. (2007), Is speech learning ‘gated’ by the social brain?. Developmental Science, 10: 110-120. https://doi.org/10.1111/j.1467-7687.2007.00572.x

References:

(1) Moreau, C. N., Joanisse, M. F., Mulgrew, J., & Batterink, L. J. (2022). No statistical learning advantage in children over adults: Evidence from behaviour and neural entrainment. Developmental Cognitive Neuroscience, 57, 101154. https://doi.org/10.1016/j.dcn.2022.101154

(2) Boucher, J., Lewis, V., & Collis, G. M. (2000). Voice processing abilities in children with autism, children with specific language impairments, and young typically developing children. Journal of Child Psychology and Psychiatry, 41(7), 847-857. https://doi.org/10.1111/1469-7610.00672

(3) Schelinski, S., Borowiak, K., & von Kriegstein, K. (2016). Temporal voice areas exist in autism spectrum disorder but are dysfunctional for voice identity recognition. Social Cognitive and Affective Neuroscience, 11(11), 1812-1822. https://doi.org/10.1093/scan/nsw089

(4) Lin, I.-F., Yamada, T., Komine, Y., Kato, N., Kato, M., & Kashino, M. (2015). Vocal identity recognition in autism spectrum disorder. PLOS ONE, 10(6), e0129451.https://doi.org/10.1371/journal.pone.0129451

**Reviewer #2 (Public review):**

Summary:

This article looks at differences in how the brain entrains to, or tracks, the rhythmic presentation of syllables and words in speech in infants at increased likelihood versus low likelihood for autism. The authors first sought to characterize how brain responses are modulated by learning the statistical probability of a given syllable following the one before it over the first two years of life. They then sought to identify at which stages of word learning infants with increased likelihood of autism showed difficulties, and whether those difficulties worsened over time. Finally, they sought to indicate whether infants' statistical learning and word learning abilities could predict later verbal skills. The authors found similar developmental trajectories of neural entrainment to syllables in infants at high and low likelihood for autism, but infants at high likelihood for autism had overall weaker syllable-level entrainment. Infants at high versus low likelihood for autism showed different developmental trajectories for word entrainment. Lower syllable entrainment in high-likelihood infants corresponded with poorer verbal outcomes, but word entrainment was not associated with verbal outcomes. Event-related potential responses to words and part words were positively associated with verbal outcomes, however, but only in low-likelihood infants.

Strengths:

Overall, the article provides rigorous statistical analysis of longitudinal EEG data to provide strong support for the claims that neural entrainment to syllable and word features of speech may be a useful marker for language development difficulties, particularly in infants at increased likelihood for neurodevelopmental disorders. The EEG data collection and preprocessing procedures are well within standards in the field. Readers should take care to note that authors indexed neural entrainment to speech using phase-locking values instead of spectral power.

Weaknesses:

While the statistical analyses are rigorous, a few of the components of the models are not clearly defined, and some corrections and thresholds for significance warrant further justification. Further, a few stimuli and participant details that could influence results are not specified. It is not clear whether all participants came from majority French-speaking families; differences in the amount of French language exposure (compared to other languages that may be spoken by a participant's family) could influence results. The standardized volume of the stimuli is also not included. As a result, readers should be encouraged to interpret that neural entrainment to speech features is likely a useful mechanism to explain differences in language development, while taking this interpretation with some caution.

We thank the reviewer for these remarks.

Regarding the amount of French exposure: while all participants were raised in primarily French-speaking environments (i.e., French as the dominant language at home and daycare), the parental questionnaire at intake indicated that 45% of the sample was exposed to additional languages, reflecting Geneva’s highly multicultural demographics. We did not quantify the extent of this exposure, which could range from very occasional exposure to situations close to true bilingualism. The structural sensitivity hypothesis (Weiss et al., 2020) posits that additional language exposure may enhance detection of statistical structures in artificial language input, even when these structures differ from those in native languages. Yet, empirical support is mixed: Yim & Rudoy (2013) found no bilingualism effect in a paradigm close to ours (triplet segmentation via auditory statistical learning, n=112 children), whereas most studies reporting bilingual advantages for statistical learning involved tasks very distinct from ours, like artificial grammar and phonotactic rule learning, or multi-cue integration for segmentation (Weiss et al., 2020).

Regarding the volume of stimuli, they were played at 50cm distance with an intensity of 75dB. Both considerations have been included in the new version of the manuscript. In general, we moved most of the figures present in Supplementary material to figure supplements to improve readability.

**Recommendations for the authors:**

**Reviewer #1 (Recommendations for the authors):**

Minor Comments:

Figure 6: The figure caption is not complete (there is no description for the right half of panel B).

We thank the reviewer for this observation, figure 6 caption has been completed.

**Reviewer #2 (Recommendations for the authors):**

Broadly speaking, I would recommend reducing the number of abbreviations in this article, and I would recommend that the authors take care as to where these abbreviations are being introduced. Many of the abbreviated terms are defined in the Materials and Methods section, which is presented after the abbreviations are used in the main results.

We acknowledge that our extensive use of abbreviations compromises the readability of the manuscript. Consequently, we have removed the following abbreviations:

- SL (replaced by statistical learning)

- LC (replaced by latent component)

- ASD (replaced by autism)

- TP (replaced by transition probability)

- MEG (replaced by magneto-encephalogram)

- MSEL (replaced by Mullen Scale of Early Learnings)

The remaining abbreviations are:

HL (high likelihood for autism), LL (low likelihood for autism), EEG (electroencephalogram), PLS-c (partial least square correlation), ERP (event-related potential), RND (random), STR (structured), BSR (bootstrap ratio), AIC (Akaike Information Criterion), PLV (phase locking value), DQ (developmental quotient), APSI (Autism Parent Screen for Infants).

Moreover, we carefully reviewed how abbreviations were introduced and identified that PLS-c, STR and RND were not defined prior to the Method section. This oversight has been corrected in the reviewed manuscript.

I would also recommend that the authors be careful with the structuring of the Introduction, particularly with their research questions and hypotheses. The article initially makes clear that the research questions are focused on the developmental trajectory of statistical learning, the levels of word learning that may differentiate high-likelihood versus low-likelihood infants, and the stability of those differences, and associations between statistical learning and various levels of word learning with verbal outcomes. The use of acoustic variability across syllables, while a valuable methodological tool, is somewhat presented as an additional research question, but not clearly stated or tested as such.

We acknowledge that the introduction (particularly the paragraph from lines 173 to 184) may have implied that speaker variability across syllables was one of our primary research aims. We clarify here that speaker variability was introduced as a mean to increase task difficulty, particularly for high-likelihood (HL) participants, with the aim of amplifying the effect sizes in our analyses.

To address this, we have removed the theoretical discussion on speaker variability in autism and typical development (lines 173–184) and explicitly stated that speaker variability was not a research question in this study. Crucially, our experimental design did not include a control condition without speaker variability, and thus we could not test its specific effects on statistical learning across age trajectories and groups.

Page 6, lines 173-176 (pages and lines refer to the reviewed uploaded manuscript): “It is worth noting, however, that our study was not designed to isolate or quantify the specific impact of speaker variability on statistical learning, as the experimental design did not include a baseline control condition omitting this acoustic variation.”

The authors do a nice job in the Materials & Methods explaining PLS-c and defining the latent components and bootstrapped ratios that will be shared in the Results. An additional brief iteration defining these statistical elements is needed at the beginning of the Results section.

We thank the reviewer for their appreciation of our Method section. We agree that an additional iteration in the result section would improve readability. We added the following paragraph at the very beginning of the Result section, briefly defining PLS-c and its main statistical output (latent components and bootstrap ratios):

Pages 6-7, lines 193-202: “Briefly, PLS-c is a data-driven multivariate modelling approach designed to identify significant patterns of electrode clusters (from a brain data matrix containing electrophysiological measures, here PLV) and their associations with “behavioral” variables (from a behavioral design matrix, here age-related parameters). Patterns of brain x behavior associations are called latent components, and their statistical significance is evaluated using permutation testing (n=1000, Bonferroni correction for number of components tested, alpha=.006). Brain and behavioral variables respective contributions to any significant latent component are tested with bootstrapping (500 random samples and replacement), with bootstrap ratios (BSR) greater than 2.3 indicating a stable contribution (for details, see the Materials and Methods section).”

(1) Page 18 Line 576. The authors need to clarify whether participants were required to be in primarily French-speaking environments and whether there was a minimum amount of French language exposure that participants were required to have if they were exposed to additional languages besides French in their everyday life.

The reviewer raises a valid concern regarding participants’ language exposure. In this study, all participants were raised in primarily French-speaking environments, with French as the dominant language at home and daycare. The parental questionnaire at intake indicated that 45% of the sample was exposed to additional languages, reflecting Geneva’s highly multicultural demographics. However, we did not quantify the extent of this exposure, which could range from very occasional exposure to situations close to true bilingualism.

The structural sensitivity hypothesis (Weiss et al., 2020) posits that additional language exposure may enhance detection of statistical structures in artificial language input, even when these structures differ from those in native languages. Yet, empirical support is mixed: Yim & Rudoy (2013) found no bilingualism effect in a paradigm close to ours (triplet segmentation via auditory statistical learning, n=112 children), whereas most studies reporting bilingual advantages for statistical learning involved tasks very distinct from ours, like artificial grammar and phonotactic rule learning, or multi-cue integration for segmentation (Weiss et al., 2020).

To include these considerations, Limitations and Material and methods sections were modified as follows:

Page 19, lines 604-607: “Second, although all participants were primarily exposed to French, we did not quantify additional language exposure, precluding any analysis of its potential moderator effects on statistical learning in our groups and age-trajectories. However, prior work has reported no effect of bilingualism on auditory triplet segmentation in children (Yim & Rudoy, 2013).”

Page 20, lines 630-631: “All participants were raised in primarily French-speaking environments, with French as the dominant language at home and daycare.”

References:

Weiss DJ, Schwob N, Lebkuecher AL. Bilingualism and statistical learning: Lessons from studies using artificial languages. Bilingualism: Language and Cognition. 2020;23(1):92-97. doi:10.1017/S1366728919000579

Yim D, Rudoy J. Implicit statistical learning and language skills in bilingual children. J Speech Lang Hear Res. 2013 Feb;56(1):310-22. doi: 10.1044/1092-4388(2012/11-0243). Epub 2012 Aug 15. PMID: 22896046.

(2) Page 18 Line 588. Further, the authors should clarify whether the 7 infants in the HL group, due to early parental concerns were defined by the 18-21-month APSI scores or by parental report prior to study enrollment.

These 7 infants were recruited based on early parental concerns prior to intake. The APSI score at 18-21 months is only reported to provide an illustration of the amount of early autistic signs that were present in these 7 infants, and to provide an estimation of their probability to develop autism later on based on Sacrey et al., 2018 longitudinal study on the APSI predictive value. We agree with the reviewer that our phrasing suggests that the APSI was used as an inclusion criterion. We rephrased the page 20 lines 642-646 as follows:

“The 7 other HL infants presented with early parental concerns for autism, based on parental report prior to enrollment. Their Autism Parent Screen for Infants (APSI) total score at their 18-21 months visit was 15.6±6.4, [8-22] range – a score greater than 8 reflecting a 63% positive predictive value for autism in HL populations.”

(3) Page 20 Line 641. The authors should specify the volume of the stimuli.

The volume of stimuli was reported in the main text (page 22, lines 695-696) as follows:

“Stimuli were played on a Bose Companion 2 Series III at a 50cm distance with an intensity of 75dB.”

(4) I'd prefer Figure 1 to be reorganized slightly - at present, the placement of the arrows explaining the analysis steps is not intuitive.

We addressed the reviewer’s comments (4) and (5) together as they both refer to Figure 1B.

(5) Page 23 lines 718-719. I think it would be helpful to explicitly define each of the interaction variables included in the behavioral design matrix. Further, this matrix should be labeled consistently in both Figure 1B and in the main text.

We refined figure 1B and its corresponding main text (in Methods section) for clarity. The arrows are now simpler and more parsimonious, labels (e.g., participant *i*, visit *n*, behavior design matrix and its parameters) are now standardized between the figure and the main text, and the interaction terms at lines 718-719 are explicitly defined.

(6) Page 23 lines 726-731: It would be helpful to know whether applying a Bonferroni correction in addition to completing permutation testing is standard when evaluating latent components derived from PLS-c. The authors should also cite justification for a bootstrap ratio cutoff of 2.3 for defining stability.

In PLS-c analyses, multiple comparisons correction across latent components and bootstrap ratio (BSR) thresholding at 2.3 are commonly adopted practices.

- Correction for multiple comparisons in PLS-c: PLS-c performs singular decomposition of the data into latent components equal in number to the variables included in the behavior design matrix (7-9 in our study, depending on the inclusion of Verbal outcome as an input variable). Each latent component’s statistical significance is assessed through permutation testing, generating a null distribution for its singular value (Krishnan et al., 2011). Given the multiple tests (one permutation test per latent component), Type I error inflation must be addressed. Recent PLS-c studies commonly applied Bonferroni correction (default procedure in the myPLS toolbox, used by Zoeller et al., 2017, and Delavari et al, 2021), though FDR correction has also been used (Lombardo et al, 2018).

- Stability threshold for bootstrap and replacement: Within each latent component, saliences’ stability (brain/behavior parameter contributions to each latent component) are evaluated using bootstrapping (Krishnan et al., 2011). The bootstrap ratio (BSR) of each parameter, calculated as the saliency divided by its bootstrap-derived standard error, functions analogously to a z-score under normality assumptions. The BSR can then be used to assess the stability of the saliency (i.e., how stable is its contribution to the latent component). BSR thresholds in the literature typically range from 1.96 to 3.0. Krishnan et al (2011) state that when BSR are “larger than 2 the corresponding saliences are considered significantly stable”. Delavari et al (2021) and our study used a 2.3 thresholding, corresponding to a 99.0% bootstrap confidence interval not crossing the zero line – roughly equivalent to a two-tailed p<.001. Lombardo et al (2018) used a looser threshold of 1.96, corresponding to a 95% confidence interval not crossing the zero line (~two-tailed p<.05), while Zöller et al (2017) used a more stringent 3.0 thresholding (~p<.001, or 99.9% confidence interval not crossing the zero line).

Thus, our application of Bonferroni correction for multiple comparisons and our 2.3 BSR threshold aligns with established conventions.

We added following lines in the manuscript:

Page 25 lines 784-785: “Bonferroni correction was applied to account for multiple comparisons across the 9 tested latent components in the PLS-c, yielding an adjusted alpha of .006 (Zoeller et al, 2017; Delavari et al, 2021).”

Page 25 lines 789-792: “BSR are analogous to Z-scores and can be used to assess the stability of the saliency. We considered BSR > 2.3 as stable, corresponding to a 99.0% bootstrap confidence interval not crossing zero – roughly equivalent to a two-tailed p<.001 (Delavari et al., 2021; Krishnan et al., 2011).”

References:

Delavari F, Sandini C, Zöller D, Mancini V, Bortolin K, Schneider M, Van De Ville D, Eliez S. Dysmaturation Observed as Altered Hippocampal Functional Connectivity at Rest Is Associated With the Emergence of Positive Psychotic Symptoms in Patients With 22q11 Deletion Syndrome. Biol Psychiatry. 2021 Jul 1;90(1):58-68. doi: 10.1016/j.biopsych.2020.12.033. Epub 2021 Jan 18. PMID: 33771350.

Lombardo, M.V., Pramparo, T., Gazestani, V. et al. Large-scale associations between the leukocyte transcriptome and BOLD responses to speech differ in autism early language outcome subtypes. Nat Neurosci 21, 1680–1688 (2018). https://doi.org/10.1038/s41593-018-0281-3

Daniela Zöller, Marie Schaer, Elisa Scariati, Maria Carmela Padula, Stephan Eliez, Dimitri Van De Ville. Disentangling resting-state BOLD variability and PCC functional connectivity in 22q11.2 deletion syndrome. NeuroImage, Volume 149, 2017, Pages 85-97, ISSN 1053-8119, https://doi.org/10.1016/j.neuroimage.2017.01.064

Anjali Krishnan, Lynne J. Williams, Anthony Randal McIntosh, Hervé Abdi, Partial Least Squares (PLS) methods for neuroimaging: A tutorial and review, NeuroImage, Volume 56, Issue 2, 2011, Pages 455-475, ISSN 1053-8119, https://doi.org/10.1016/j.neuroimage.2010.07.034

(7) I have a few minor grammar/formatting recommendations for the authors as well:

(a) Should the Geneva Autism Cohort be capitalized? At present, it is not.

We agree with the reviewer’s suggestion, and we capitalized the Geneva Autism Cohort in the main text (page 18, line 571)

(b) Page 24, line 750. Do the authors mean that the data was re-referenced to average?

The preprocessed data is not average-referenced (see section Data pre-processing). Therefore, both for neural entrainment computation and ERPs, the data were average-referenced.

(c) It would be nice to have a figure of the actual ERP for each condition and age group.

We agree that PLS-c can be difficult to interpret without the raw actual ERPs on which it was modelled. We direct the reviewer to supplementary figure S6 at page 59, which displays the raw ERPs for each condition (part-word, word, and their subtraction) per age group. Supplementary figures S7-8 at pages 60-61 further illustrate topographical ERPs for each group (high and low likelihood for autism). We deemed these figures too extensive for the main text. Instead, the most relevant ERP topographies are presented in Figures 4-6 to facilitate PLS-c interpretation.
